# The Ankle Recovery Trial (ART): clinical outcomes and patient experience of a pragmatic multicentre RCT comparing cast with removable boot for early mobilization after ankle fracture surgical fixation

**DOI:** 10.1302/2633-1462.611.BJO-2025-0108.R1

**Published:** 2025-11-10

**Authors:** Rachel Martin, Sharon Docherty, Vanessa Heaslip, Helen Allen, Lee W. Tbaily, Christopher J. Hayward, Elsa M. R. Marques, Anuraag Sangar

**Affiliations:** 1 University Hospitals Dorset NHS Foundation Trust, Longfleet Road, Poole Hospital, Poole, UK; 2 Faculty of Health and Social Sciences, Bournemouth University, Bournemouth, UK; 3 University of Salford, Salford, UK; 4 Department of Social Work, University of Stavanger, Stavanger, Norway; 5 Exeter Clinical Trials Unit, College House, St Luke’s Campus, Exeter, UK; 6 Musculoskeletal Research Unit, Bristol Medical School, Translational Health Sciences, Bristol, UK

**Keywords:** Ankle fracture, Early mobilization, Plaster cast, Air boot, RCT, ankles, functional outcomes, dorsiflexion, ankle fracture fixation, deep vein thrombosis (DVT), wound complications, Ankle Symptom

## Abstract

**Aims:**

This study compares functional outcomes and patient experience between cast immobilization and early mobilization in a removable boot after ankle fracture fixation, with early weightbearing encouraged in both groups.

**Methods:**

This pragmatic multicentre randomized controlled trial with qualitative component and economic evaluation was conducted across eight UK NHS hospitals. Adults with acute ankle fractures were randomized to receive a plaster cast or removable support boot two weeks post-surgery. The primary outcome was ankle function measured by the Olerud and Molander Ankle Symptom Score (OMAS) seven weeks post-surgery. Secondary outcomes included function at 12 weeks, mechanistic measures, quality of life, complications, and resource use. Subgroup analyses included fracture complexity and age. Patients’ views on both treatments were collected through semi-structured telephone interviews.

**Results:**

In total, 243 participants consented to be randomized (120 cast; 123 boot), of whom 173 (71.2%) completed the primary outcome. The mean difference in OMAS at seven weeks between groups was 4.9 points favouring the boot (95% CI -1.0 to 10.7), which is below the minimal clinically important difference, and failed to detect a difference between groups. Boot participants had better dorsiflexion, particularly those with comminuted fractures, and better plantarflexion, particularly older patients. Complication rates were low, albeit higher in the boot group (cast eight/112; boot 18/117); all were minor, except one case of deep vein thrombosis in the boot group. Overall, we found low wound complication rates (7%). There were no differences for all other secondary measures. Patients expressed preference for boots at randomization, point of withdrawal from the trial, and during interviews.

**Conclusion:**

Patients managed in casts and boots had similar functional outcomes following ankle fracture fixation. Boots provided improved dorsiflexion and plantarflexion for some subgroups, but higher complication rates. Treatment modality decisions could therefore be informed by individual patient preference.

Cite this article: *Bone Jt Open* 2025;6(11):1416–1424.

## Introduction

Ankle fractures are a common injury leading to approximately 19,000 UK hospital admissions annually.^1^ Causing short and long-term disability and pain, they frequently result in many weeks off work with subsequent economic consequences.^2^ Over half of patients still experience symptoms such as gait abnormalities, inability to return to previous activity, and mental health issues up to three years post-injury.^3-5^ It is important for postoperative management to maximize recovery of function and reduce the risk of long-term functional and psychological consequences.

Traditionally, patients who have undergone ankle fracture fixation have been managed in a plaster cast non-weightbearing for several weeks. Casts provide maximum support but prolonged immobilization can cause joint stiffness, muscle atrophy, and deep vein thrombosis (DVT).^6-8^ Early weightbearing has been shown to be safe with minimal risk of metalwork or fracture displacement.^9-12^ However, evidence on early ankle mobilization remains conflicting. While it has been shown to improve range of motion (ROM) and reduce swelling and muscle atrophy, these benefits are short-lived.^2,8^ Some studies report earlier return to work, improved patient satisfaction, and better ankle function with early mobilization.^8,13^ However, high wound complication rates have been associated with immediate mobilization,^14^ with a lower incidence when mobilization is delayed until after primary wound healing.^8^ The most recent Cochrane review^15^ concluded that functional bracing may improve function, pain, and ankle movement but should be balanced against the increased incidence of adverse events. The review called for better designed trials with adequate numbers.

The Ankle Recovery Trial (ART) was designed to assess the effectiveness and cost-effectiveness of comparing early mobilization in a removable boot with cast immobilization following ankle fracture fixation, where early weightbearing was encouraged in both groups. This paper reports the clinical effectiveness results of functional outcomes and patient experience reported in the nested qualitative study. The economic evaluation reporting costs, quality of life, and cost-effectiveness is published in a separate economic evaluation paper.^16^

## Methods

ART was a pragmatic multicentre randomized controlled trial (RCT) with an embedded qualitative component and health economic evaluation conducted across eight UK NHS secondary care trusts. An independent steering committee oversaw the study and approved the analysis plan. The study (ISRCTN 15497399) was given ethical approval by South Central Hampshire A Research Ethics Committee (14/SC/1409).

### Participant characteristics

Patients eligible for the study were aged over 16 years and undergoing open reduction and internal fixation (ORIF) for an unstable ankle fracture. Exclusion criteria were open ankle fracture, concern about quality of fixation or wound integrity, requiring further stabilization (e.g. syndesmosis), active leg ulceration, poor skin condition, serious concomitant disease, diabetic or other sensory neuropathy, nonambulatory prior to injury, inability to complete outcome questionnaires, enrolment in other research which may confound data, and concomitant injuries which may affect rehabilitation.

**Fig. 1:**
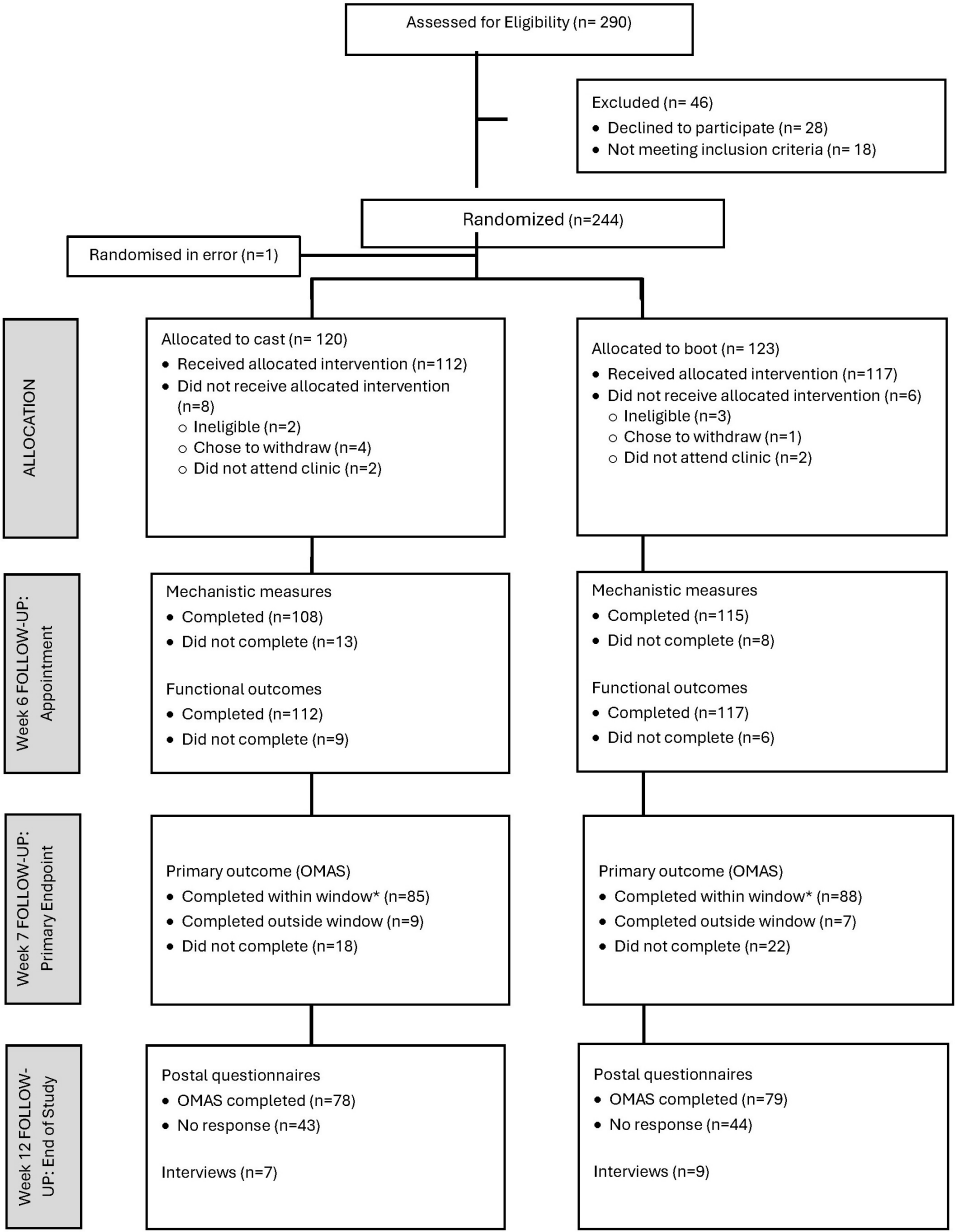
Flow of participants through the stages of the trial. *The window for a valid response for the primary outcome was three to ten days after the week six follow-up appointment. OMAS, Olerud-Molander Ankle Symptom Score.

Between July 2015 and September 2018, eight NHS sites screened 1,404 patients. In all, 290 patients registered for the trial and were assessed for eligibility (Figure 1), 18 of whom were ineligible. Of those who were eligible, 28 declined to participate. Reasons included treatment preference (one for cast, 12 for boot); unwilling to be randomized with no stated preference (n = four); concern over early weightbearing (n = one); and no reason given (n = ten).

Baseline characteristics (including fracture complexity, Weber fracture classification, and medial malleolar involvement) were well-balanced between groups (Table I).

**Table I.** Patient characteristics by treatment group.

Variable	Cast	Boot
Total, n	120	123
Mean age, yrs (SD)	47.7 (17.1)	48.8 (15.7)
Male sex, n (%)	49 (41.2)	46 (37.4)
Right side, n (%)	66 (55.9)	66 (54.1)
Mean BMI, kg/m^2^ (SD)	27.4 (5.5)	28.3 (5.8)
Not living alone prior to injury, n (%)	95 (84.1)	96 (79.3)
In paid employment prior to injury, n (%)	72 (60.0)	80 (65.0)
Driving prior to injury, n (%)	66 (55.9)	62 (50.4)
**Fracture complexity, n (%)**		
Simple	99 (83.9)	101 (82.8)
Comminuted	19 (16.1)	21 (17.2)
**Weber fracture classification, n (%)**		
A	0 (0.0)	1 (1.0)
B	94 (94.0)	96 (96.0)
C	6 (6.0)	3 (3.0)
Medial malleolar involvement in fracture, n (%)	56 (47.5)	58 (47.5)
Mean from injury to surgery, days (SD)	6.8 (5.2)	6.5 (5.7)
Mean pre-injury OMAS (SD)	96.0 (9.7)	96.9 (8.2)
Mean EQ-5D Health Today (SD)	72.0 (17.6)	72.0 (18.0)

EQ-5D Health Today, health barometer of the EuroQol five-dimension five-level questionnaire; OMAS, Olerud-Molander Ankle Score.

### Randomization and blinding

After baseline data collection at the two-week postoperative assessment, participants were randomized in a one:one ratio to receive either a plaster cast or removable support boot. Randomization was computer-generated and conducted in random block sizes of two, four, six, or eight, stratified by participating site. It was not possible to blind participants and clinicians to allocation.

### Interventions

Following surgery, the ankle was immobilized in a backslab until the patient’s routine follow-up appointment, two weeks later. After backslab removal and wound checks, participants received their allocated intervention. The control group received a standard below-knee plaster cast and shoe. The intervention group received a removable fixed-angle walking boot with rigid outer sections and at least two inflatable air chambers.

All participants were given an instruction leaflet specific to their treatment group with exercises taught by a physiotherapist. Participants were encouraged to complete exercises as often as pain allowed (≥ three times a day), advised on gait re-education with crutches, and to progress weightbearing as able.

At the six-week postoperative appointment, participants had their cast or boot removed and undertook routine clinical assessments and radiographs to guide subsequent management. Most patients were expected to dispense of the cast or boot at this point. However, the decision to extend the duration in the cast/boot, refer to physiotherapy, or arrange further clinical review was left to the clinicians’ discretion.

### Outcome measures

Follow-up was in person with a member of the research team at six weeks, then via postal/online/telephone questionnaires at seven and 12 weeks post-surgery.

The primary outcome was ankle function measured by the Olerud and Molander Ankle Symptom Score (OMAS),^17^ consisting of domains covering pain, stiffness, swelling, stair climbing, running, jumping, squatting, supports, and activities of daily living. Possible scores range from 0 to 100 with higher scores indicating better function. The seven-week timepoint was chosen to allow participants to adjust to cast/boot removal for a more accurate assessment of functional recovery. This was repeated at week 12 as a secondary outcome measure. Overall perception of the impact of ankle injury on daily activities (0 to 10 scale, ranging from no effect to completely prevented daily activities) was collected at six and 12 weeks.

Secondary mechanistic measures included ankle ROM (dorsiflexion, plantarflexion, inversion, and eversion), ankle (‘figure of 8’ method),^18^ and calf circumference measured by a physiotherapist at six weeks.

Other functional outcomes included weightbearing status and use of walking aids at weeks six and 12, as well as number of days to return to previous levels of employment and driving. Complications and serious adverse events were reported at the week six visit and via medical notes at 12 weeks. Resource use, and EuroQol five-dimension five-level questionnaire (EQ-5D-5L)^19^ were the primary economic measure of health benefit.

### Patient experience

Approximately 12 weeks post-surgery, semi-structured telephone interviews were conducted by a qualitative researcher (VH, HA) with a subset of participants to explore their experiences of the holistic impact of the cast/boot on their everyday lives and that of their families. The sample was selected to encompass a balance of treatment options, sex, age, hospital site, and pre-injury OMAS scores. Interviews were transcribed verbatim and analyzed using thematic analysis.^20^ Interviews were analyzed independently, and themes identified from the data were agreed between two researchers (VH, HA).

### Sample size

Sample size was calculated using the OMAS at seven weeks post-surgery as the primary outcome measure. Based on an independent-samples *t*-test with two-sided significance level (α) of 0.05 and 90% power (β), SD of 21.9, and mean between group difference of 10 points on the OMAS (minimum clinically important difference),^5^ a total sample size of 204 (102 per group) was required. The sample size was inflated by 20% to accommodate for non-responders and missing data, giving a target of 246 participants.

### Statistical analysis

Analyses were blinded to allocation and performed on an intention-to-treat basis. There was no imputation for missing data. Multiple regression was used to compare OMAS at seven weeks between cast and boot groups. Study site, age group (≤ 64 years or ≥ 65 years), and fracture complexity (simple versus comminuted) were prespecified factors included as fixed effects. Multiple regression was also used to investigate differences between groups in the other continuous outcomes measured at six weeks (active ROM, circumferential measurements); and 12 weeks (OMAS, impact on daily activities). Complications were reported using odds ratios and tested with Fisher’s exact test.

Sensitivity analyses included a per-protocol analysis excluding patients who changed from allocated intervention or reported not doing prescribed exercises at least once per day, and inclusion of those completing the seven-week OMAS outside the specified three to ten day window. Treatment effects were reported with mean and 95% CIs alongside a prespecified significance level of p < 0.05. Analyses were conducted using IBM SPSS Statistics for Windows v. 26 (IBM, USA).

## Results

One person was randomized in error having not provided consent and was excluded from all analyses. 243 participants consented to be randomized (120 cast; 123 boot) and provided baseline data. Most participants (94%) received the allocated treatment, six did not receive the boot while nine did not receive the cast (three cited boot preference). Exercise compliance was high and did not differ significantly between groups; 93% of participants completed exercises at least once a day, while 56% completed them at least three times a day (Supplementary Material).

### Patient-reported outcomes

**Fig. 2:**
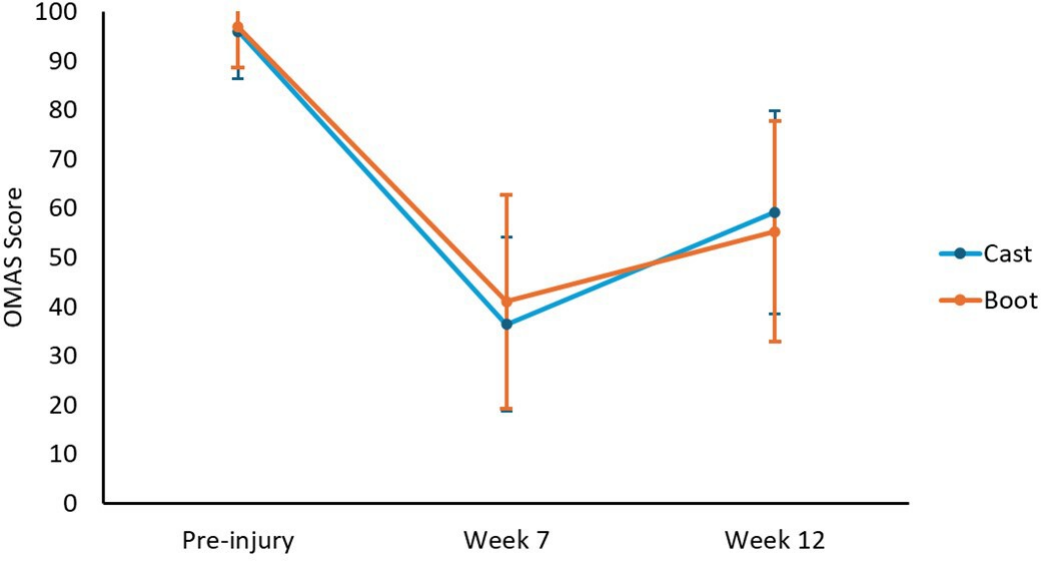
Comparison of mean Olerud and Molander Ankle Score measures between cast and boot groups. Possible scores range from 0 to 100, with higher scores indicating better function. SDs are displayed as error bars. Week seven was the primary endpoint.

**Fig. 3:**
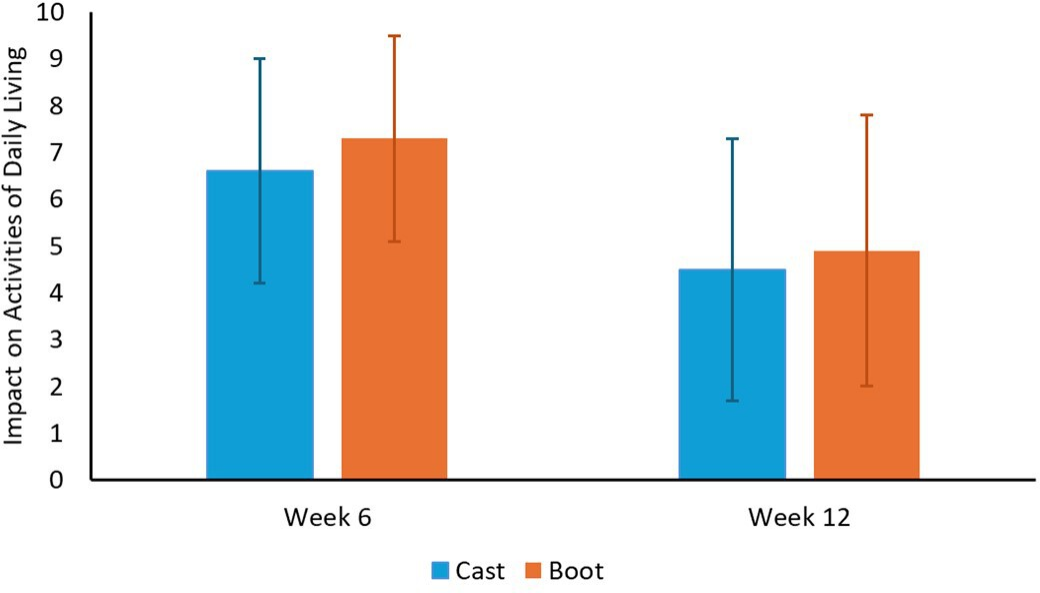
Comparison of mean impact on activities of daily living score between cast and boot groups (0 to 10 scale, ranging from no effect to completely prevented daily activities). SDs are displayed as error bars.

Of 243 participants, 173 (71.2%) completed the OMAS at week seven (primary outcome) within the specified time window. Scores ranged from 5 to 90 and did not differ significantly between boot and cast groups (Table II, Figure 2 and Figure 3) at weeks seven and 12. The boot group reported greater negative impact on daily activities at week six, but by week 12 there was no longer evidence of an effect (Table II and Figure 3).

Results were robust to all sensitivity analyses (Supplementary Material).

**Table II.** Patient-reported outcomes of ankle function and health by allocated treatment.

Outcome	Cast		Boot		Treatment effect	
**OMAS**	**n**	**Mean score (SD)**	**n**	**Mean score (SD)**	Adjusted between-group differences (95% CI)*	**p-value†**
Week 7 OMAS‡	85	36.4 (17.7)	88	41.0 (21.8)	4.9 (-1.0 to 10.7)	0.100
Week 12 OMAS	78	59.2 (20.6)	79	55.3 (22.4)	-2.3 (-9.0 to 4.3)	0.490
**Impact on everyday activities**						
Week 6 rating	99	6.6 (2.4)	103	7.3 (2.2)	0.7 (0.1 to 1.3)	0.029
Week 12 rating	76	4.5 (2.8)	74	4.9 (2.9)	0.2 (-0.7 to 1.2)	0.620

* Mean difference between groups is adjusted for site (fixed effect), age group ( ≤ 64 years or ≥ 65 years), and fracture complexity (simple or comminuted).

† Multiple regression.

‡ Primary outcome.

OMAS, Olerud-Molander Ankle Score.

### Mechanistic measurements

Table III reports the difference between each participant’s non-injured and injured ankles in ROM ankle circumference, and calf circumference between groups at week six. Smaller differences were observed between injured and non-injured ankles for participants in the boot group for dorsiflexion and plantarflexion. There was some evidence for interactions between fracture complexity and treatment arm for dorsiflexion (mean -3.7, 95% CI -7.4 to -0.04; p = 0.048) indicating a larger effect in participants with comminuted versus simple fractures. For plantarflexion, participants aged ≥ 65 years experienced a greater effect (mean -8.0, 95% CI -15.0 to -0.9; p = 0.027) than those aged ≤ 64 years. There were no significant differences in eversion, inversion, ankle or calf circumference between groups.

**Table III.** Ankle range of motion and circumferential measurements at week six; physiotherapy appointment by allocated treatment.

	Cast		Boot		Treatment effect	
Outcome	n	Mean (SD)	n	Mean (SD)	Adjusted between-group differences (95% CI)^*^	p-value†
**Ankle range, °‡**						
Dorsiflexion	106	8.2 (6.1)	114	6.5 (4.4)	-1.7 (-3.1 to 0.3)	0.016
Plantarflexion	108	20.8 (11.0)	115	16.4 (10.8)	-4.8 (-7.5 to –2.0)	0.001
Inversion	108	17.1 (9.5)	115	15.0 (8.7)	-2.3 (-4.7 to 0.02)	0.052
Eversion	108	8.1 (6.6)	115	7.5 (6.4)	-0.7 (-2.4 to 1.0)	0.420
Ankle circumference, cm§	108	-2.80 (3.60)	115	-2.95 (1.97)	-0.10 (-0.8 to 0.63)	0.790
Calf circumference, cm¶	108	2.06 (3.85)	114	2.11 (1.71)	0.04 (-0.74 to 0.82)	0.920

* Mean difference between groups is adjusted for site (fixed effect), age group (≤ 64 years or ≥ 65 years), and fracture complexity (simple or comminuted).

† Multiple regression

‡ All measurements are difference between non-injured and injured ankle with larger differences indicating poorer movement in injured ankle.

§ Difference between non-injured and injured ankle with smaller (negative) differences indicating swelling in the injured ankle.

¶ Difference between calf of non-injured and injured limb with greater (positive) differences indicating calf atrophy on the injured side.

### Secondary functional outcomes

At week six, 197 participants (86.0%) were fully weightbearing, with 85 participants (37.3%) not using walking aids (Table IV). By week 12, this increased to 135 (95.1%) and 112 (76.7%) participants, respectively; 70 (60.9%) participants returned to full preinjury work duties; and 106 (82.8%) participants returned to driving. There were no significant differences between groups in these measures at any timepoint.

**Table IV.** Secondary functional outcomes by allocated treatment.

Outcome	Cast		Boot		Treatment effect	
**Full weightbearing**	**n**	**Events, n (%**)	**n**	**Events, n (%**)	**Adjusted OR (95% CI**)^*^	**p-value†**
Week 6	112	98 (87.5)	117	99 (84.6)	0.75 (0.34 to 1.65)	0.470
Week 12	73	72 (98.6)	69	63 (91.3)	0.17 (0.02 to 1.60)	0.122
**No walking aids**						
Week 6	111	38 (34.2)	117	47 (40.2)	0.71 (0.4 to 1.28)	0.260
Week 12	75	63 (84.0)	71	49 (69.0)	2.33 (0.98 to 5.54)	0.056
**Return to employment**		**Median, % returned**		**Median, % returned**	**Adjusted HR (95% CI)** ^*^	
Full pre-injury duties, days	56	40.0 (60.7)	59	36.0 (61.0)	0.92 (0.56 to 1.51)	0.750
**Return to driving**						
Pre-injury driving status, days	66	42.0 (84.8)	62	46.0 (80.6)	0.97 (0.65 to 1.45)	0.900

* Adjusted for site (fixed effect), age group ( ≤ 64 years or ≥ 65 years), and fracture complexity (simple or comminuted).

† Logistic regression.

HR, hazard ratio; OR, odds ratio.

### Complications

The number of complications experienced by participants was low but more frequently reported in the boot group (cast 8/112, boot: 18/117; OR 2.36; 95% CI 0.15 to 1.08; p = 0.061), most notably for wound-related issues (Table V). Apart from one reported DVT in the boot group, which resolved without hospital admission, complications were mostly minor in both groups. Wound breakdowns healed with simple dressings and infections were superficial, resolving with oral antibiotics.

**Table V.** Number of treatment related complications and adverse incidents by allocated treatment.

	Cast		Boot	
**Complication**	**n**	**Events, n (%**)	**n**	**Events, n (%**)
Wound complications	112		117	
Breakdown		0 (0.0)		7 (6.0)
Minor infection		2 (1.8)		7 (6.0)
Blisters, minor	112	6 (5.4)	117	4 (3.4)
Pressure sore, stage 1	112	0 (0.0)	117	1 (0.9)
Nerve injury (sensory)	112	3 (2.7)	117	2 (1.7)
Deep vein thrombosis	112	0 (0.0)	117	1 (0.9)
Pain	112	1 (0.9)	117	3 (2.6)

Includes all with complications data at four weeks; assumes zero complications in those with missing data (eight cast, six boot) at ten weeks.

### Patient experience

Overall, 16 participants (seven cast, nine boot) were interviewed, comprising six males and ten females with a mean age of 49.2 years (24 to 77 years). All interviewees confirmed the significant impact the fracture had on their lives. Physically, this manifested in reduced sleep and inability to undertake activities of daily living (e.g. showering and mobilizing). For those in casts, these issues were increased due to the weight of the plaster and difficulties getting comfortable, whereas many of those in boots removed them at night, aiding their sleep.

“It would get swollen and quite tight and uncomfortable so I would try to keep it elevated while I was sleeping.” *(Female, 44, cast)*

“After a fortnight, I didn’t need to sleep with it all, I just had pillows on my bed and that made a huge difference in terms of comfort to my hips and thigh.” *(Female, 69, boot)*

Psychologically, participants described feeling vulnerable and reliant upon others, which impacted upon their mood. Ten participants described low mood during their recovery (six cast; four boot); additionally, four participants (cast) felt frustrated due to immobility.

“I felt down a little bit because I am normally a really active person and now all of a sudden…I couldn’t just go for a run or… go and play football and that had a negative effect.” *(Male, 24, boot)*

“I am always the one doing everything, all of a sudden, I couldn’t and I got so bored, frustrated and angry…. [cast] gave me severe depression.” *(Female, 54, cast)*

Having a boot appeared to mitigate this as eight participants with boots reported increased confidence in walking and undertaking activities, which improved their mood. Participants expressed they felt more in control, more independent, and liked that they were able to adjust the boot to monitor their own wound healing. Participants also described feeling able to participate in their recovery through moisturising their leg and self-managing swelling.

“I was able to wash my leg and I could moisture it, using the bio-oil on the scars which I think has helped really well, because everyone saying how well my scars have healed.” *(Female, 46, boot)*

“You feel you weren’t trapped… if you couldn’t take the plaster off you would think ‘oh this damned thing’, but with the boot you think ‘oh I’ll just take it off for a minute because my foot needs a bit of air getting to it.’” *(Male, 51, boot)*

“[Boot] definitely gave me more confidence and manoeuvrability …and I felt that I was actually making progress, you know every little milestone with the boot on you know it just made me think I am going to get better.” *(Female, 77, boot)*

## Discussion

This large, multicentre RCT was heavily informed through consultation with patient groups (particularly the choice of primary outcome), including embedded qualitative and economic evaluation components to allow a more in-depth understanding of the patient experience and value for money. We found no meaningful difference in ankle function between groups at seven or 12 weeks postoperatively. At six weeks, boot participants had better dorsiflexion, especially those with comminuted fractures. They also had better plantarflexion, particularly older patients. However, wound complications were more common in the boot group, plus one case of DVT, which resolved with basic treatment. Patients expressed a stronger preference for boots, reporting that they felt more in control of their recovery and empowered to resume social and family activities.

Our findings for the OMAS score are in line with other studies, where no meaningful difference between groups was found.^13,21-24^ However, during interviews, all participants in the cast group reported increased difficulty undertaking activities of daily living compared to only one boot participant. This finding was not reflected in the OMAS scores or impact on everyday activities scores. While OMAS is a widely used outcome measure in ankle studies, it may not be sensitive enough to detect the differences in function most important to patients in the early recovery stage.

Our finding of superior ROM in the boot group is consistent with previous literature.^2,15^ Dehghan et al^6^ (n = 110) compared non-weightbearing casts with weightbearing boots and reported better outcomes in ROM and OMAS at six weeks; however, differences in OMAS had diminished by 12 weeks. Unlike Honigmann et al,^21^ who reported earlier return to work in a fully weightbearing vacuum boot compared to partial weightbearing in a bandage, we found no significant difference in impact on everyday activities, time to return to work, or driving between groups. This suggests the ability to weightbear may be more important than ROM with regards to differences in OMAS and other functional outcomes.

Participants in boots reported greater comfort, stronger sense of control, and enhanced engagement in their recovery; interviews revealed they appreciated the ability to monitor wound hygiene and healing. However, this convenience may compromise outcomes as ability to remove the boot may have affected adherence to medical guidance, reflected in a higher incidence of wound infections in this group. This highlights the need for clinicians to provide targeted education on self-management and the risks of non-compliance,^25^ ensuring the advantages of comfort and autonomy do not come at the cost of preventable complications.

Improved comfort may also support psychological readiness for recovery, which is linked to better rehabilitation outcomes.^26^ When appropriately guided, psychologically well-adjusted patients are more likely to adhere to clinical recommendations. Furthermore, psychological distress may significantly impair bone healing and heighten pain perception.^27^

A patient-centred approach requires more than accommodating preferences; it involves understanding patients' beliefs, expectations, and readiness for self-care. In turn, clinicians must communicate the consequences of non-compliance clearly and adopt a model of care that fosters shared decision-making and therapeutic alliance.^28,29^

These findings offer practical guidance for clinicians balancing patient preference with medical outcomes. While many studies have documented the psychological burden of immobility and fracture-related uncertainty, clinical judgement remains critical, particularly when assessing health literacy or cognitive ability. In patients unlikely to adhere to boot protocols, a cast may be more appropriate to safeguard tissue healing and minimize complications.

These findings also complement the economic evaluation whereby no differences in quality of life between groups were found at 12 weeks, and the boot was slightly more expensive to the health and social care payer by £88 (95% CI £22 to £155); but when including all patients’ private expenses, productivity losses and informal care, the boot provided, on average, a saving of £676 per patient, albeit with a wide CI crossing the null (95% CI -£337 to £1,689).^15^

This trial used a pragmatic approach to boot selection and postoperative management, allowing clinicians to follow local NHS practices. Although these data were collected several years ago, the results remain relevant and generalizable to current NHS practices.

Another key feature of the trial was early weightbearing in both the plaster and boot groups allowing a more accurate evaluation of the impact of early mobilization. This is especially important in elderly patients who are unlikely to manage non-weightbearing and may otherwise have lengthy inpatient stays and increased risk of complications. While other studies have identified not recording exercise frequency as a potential limitation,^24^ we recorded high levels of compliance with no difference between groups (Supplementary Material).

A limitation of the study was a higher than anticipated loss to follow-up rate (29% at week seven), which could suggest that the study was underpowered to find a difference between the groups. However, the observed SD for the primary outcome was lower than estimated (20.0 points vs 21.9 points) and a post-hoc calculation confirmed 90% power with the achieved sample size.

Due to the relatively short follow-up period, this study was unlikely to capture differences in rates of reoperation, longer-term complications, or return to preinjury function. However, Haque et al^24^ reported that outcomes remained similar between groups after two years.

In conclusion, this trial found no significant difference in functional outcomes between treatment with plaster cast compared with removable boot after ankle surgery when patients were weightbearing early post-surgery. The boot group reported better dorsiflexion for those with comminuted fractures and better plantarflexion for older participants, but it was also associated with more complications, particularly wound complications. Patients expressed a stronger preference for boots and a more positive experience.

Early weightbearing in either a removable boot or plaster cast following ankle fracture fixation produced similar functional outcomes. Therefore, treatment modality decisions could be informed by clinical circumstances and patient choice.

**Take home message**

- Early weightbearing in either a removable boot or plaster cast following ankle fracture fixation produced similar functional outcomes.

- Therefore, treatment modality decisions could be informed by clinical circumstances and patient choice.

## Data Availability

The data that support the findings for this study are available to other researchers from the corresponding author upon reasonable request.
